# Effectiveness and safety of artemether–lumefantrine versus artesunate–amodiaquine for unsupervised treatment of uncomplicated falciparum malaria in patients of all age groups in Nanoro, Burkina Faso: a randomized open label trial

**DOI:** 10.1186/s12936-015-0843-8

**Published:** 2015-08-20

**Authors:** Paul Sondo, Karim Derra, Seydou Diallo-Nakanabo, Zekiba Tarnagda, Odile Zampa, Adama Kazienga, Innocent Valea, Hermann Sorgho, Ellis Owusu-Dabo, Jean-Bosco Ouedraogo, Tinga Robert Guiguemde, Halidou Tinto

**Affiliations:** IRSS, Clinical Research Unit of Nanoro (CRUN), CMA Saint Camille of Nanoro, BP 218 Ouagadougou CMS 11, Nanoro, Burkina Faso; Centre Muraz of Bobo-Dioulasso, Bobo-Dioulasso, Burkina Faso; Kumasi Center for Collaborative Research in Tropical Medicine (KCCR), Kumasi, Ghana

**Keywords:** Effectiveness, Artesunate–amodiaquine, Artemether–lumefantrine, Unsupervised-treatment, Malaria

## Abstract

**Background:**

Several studies have reported high efficacy and safety of artemisinin-based combination therapy (ACT) mostly under strict supervision of drug intake and limited to children less than 5 years of age. Patients over 5 years of age are usually not involved in such studies. Thus, the findings do not fully reflect the reality in the field. This study aimed to assess the effectiveness and safety of ACT in routine treatment of uncomplicated malaria among patients of all age groups in Nanoro, Burkina Faso.

**Methods:**

A randomized open label trial comparing artesunate–amodiaquine (ASAQ) and artemether–lumefantrine (AL) was carried out from September 2010 to October 2012 at two primary health centres (Nanoro and Nazoanga) of Nanoro health district. A total of 680 patients were randomized to receive either ASAQ or AL without any distinction by age. Drug intake was not supervised as pertains in routine practice in the field. Patients or their parents/guardians were advised on the time and mode of administration for the 3 days treatment unobserved at home. Follow-up visits were performed on days 3, 7, 14, 21, and 28 to evaluate clinical and parasitological resolution of their malaria episode as well as adverse events. PCR genotyping of merozoite surface proteins 1 and 2 (*msp*-*1*, *msp*-*2*) was used to differentiate recrudescence and new infection.

**Results:**

By day 28, the PCR corrected adequate clinical and parasitological response was 84.1 and 77.8 % respectively for ASAQ and AL. The cure rate was higher in older patients than in children under 5 years old. The risk of re-infection by day 28 was higher in AL treated patients compared with those receiving ASAQ (p < 0.00001). Both AL and ASAQ treatments were well tolerated.

**Conclusion:**

This study shows a lowering of the efficacy when drug intake is not directly supervised. This is worrying as both rates are lower than the critical threshold of 90 % required by the WHO to recommend the use of an anti-malarial drug in a treatment policy.

Trial registration: NCT01232530

## Background

Malaria remains the main cause of morbidity and mortality in Burkina Faso. Based on the recommendation of the World Health Organization, artemisinin-based combination therapy (ACT) was adopted by the national malaria control programme (NMCP) for the treatment of uncomplicated malaria since 2005. The two combinations adopted in the country as first-line regimen are artemether–lumefantrine (AL) and artesunate–amodiaquine (ASAQ) [1, 2]. Globally, several efficacy and effectiveness studies have demonstrated their high efficacy and safety for the treatment of uncomplicated *Plasmodium falciparum* malaria [3–5].

However, in most of these studies, drug intake was supervised by a study team and treatment was strictly administered in accordance with manufacturer’s recommendations. Yet, in poor resource settings with high malaria morbidity, strict adherence to these conditions is often not observed in real life settings. A recent study conducted in this study area has already shown a lowering of the cure rate of ACT in real life comparative to the other efficacy studies [6]. In addition, most of those studies were limited to children under five and the older populations are typically not involved. Despite the upheld notion that adults are immune-protected, behaviour towards the treatment, such as not completing treatment regiments, could be higher in adults and this could negatively influence treatment outcomes. Therefore, an assessment of treatment outcomes that would fully reflects the reality in the field has to involve all age groups in the population. Moreover, the best indicator for safety could be obtained from adults since children are challenged to make recalls on safety information. It is based on these compelling reasons that this study was carried out in order to assess the effectiveness and safety of the two recommended forms of ACT in Burkina Faso as they are used routinely in treating uncomplicated malaria in all age groups in the country.

## Methods

### Study area

The study was carried out from September 2010 to October 2012 at two primary health centers (Nanoro and Nazoanga) of Nanoro health district (NHD) in Burkina Faso. Nanoro is situated at about 85 km from Ouagadougou, the capital city of the country. It is classified as hyperendemic and malaria transmission is seasonal with a peak at rainy season that lasts usually from June–July to October–November [7]. The entomological inoculation rate is estimated at 50–60 infective bites/person/year and the commonest vectors are *Anopheles gambiae*, *Anopheles funestus* and *Anopheles arabiensis* (A. Diabate, personal communication)*. Plasmodium falciparum* is the most prevalent malaria parasite. The predominant ethnic group is Mossi, minorities are Gourounsi and Fulani. The majority of the population practice subsistence farming [8].

### Study participants and inclusion criteria

Patients presenting at the two health facilities with symptoms or signs suggestive of malaria were reviewed by study nurses without any age distinction. The inclusion criteria were: (a) fever or history of fever within the previous 24 h, (b) mono infection with *P. falciparum*, (c) parasites density comprised between 2000 and 200,000 asexual forms per microlitre of blood, (d) haemoglobin >5 g/dL. Patients were excluded from the study if they presented (a) danger signs (unable to drink or breast-feed; vomiting more than twice in 24 h; recent history of convulsions; unconsciousness or unable to sit or stand), (b) severe malaria, (c) severe malnutrition (defined as weight for height <70 % of the median NCHS/WHO reference), (d) a documented history of adequate malaria treatment in the preceding 2 weeks, (e) any evidence of chronic disease or of a concurrent non-malarial febrile illness, (f) history of serious side effects with the study drugs, (g) a reported pregnancy for child bearing age women and patient or his parent/guardians unwillingness to participate.

### Study procedures

Patients were randomly assigned to receive either AL (Coartem^®^, Novartis) or ASAQ (Winthrop, sanofi aventis). A computer-generated randomization list provided by the site quality assurance manager was used for treatment allocation. Treatment was administered according to the national algorithm for malaria cases management and was strictly adhered to. In ASAQ group, treatment was administered once daily, at the standard dose as follows: 4.5 to <9 kg, 1 tablet 25/67.5 mg; 9 to <18 kg, 1 tablet 50/135 mg; 18 to <36 kg, 1 tablet 100/270 mg; ≥36 kg, 2 tablets 100/270 mg. In AL group, treatment was given twice daily according to the body weight as follows: 5–14 kg, one tablet per dose; 15–24 kg, two tablets per dose; 25–34 kg, three tablets per dose and adult, four tablets per dose. Patients (adults) or their parents/guardians (children) were advised of the time and mode of administration for the 3 days treatment taken at home unobserved. Patients and or their parents/guardians were advised to administer AL with fat containing food and were asked to come back (with their child) for scheduled visits on days 3, 7, 14, 21, and 28, or if the participant was sick between visits (unscheduled visit). At each visit a physical examination was performed. Blood sample was taken from the finger to prepare thick/thin film, photometric measurement of haemoglobin (HemoCue^®^ 301^+^) and spotted onto filter paper (Whatman 3MM, Maidstone, UK) for later molecular analysis. For identification and reporting of AEs, patients were assessed at each visit according to a standardized checklist and the information was recorded on the case record form (CRF). A severity grading scale, based on the toxicity grading scales developed by the WHO was used to grade severity of all reported adverse events (AEs) and clinical examination findings. All AEs were catalogued based on their frequency, severity, and relationship with AL and ASAQ treatment [9].

### Microscopic examination

Blood smears were read in the field at each health facility by light microscopy after they have been stained with 3 % Giemsa for 30 min. Parasite density was determined by counting the number of asexual parasites per 200 white blood cells, and calculated per micro liter of blood by assuming the white blood cells at 8000/µL. A smear was declared negative when the examination of 100 thick-film fields did not reveal the presence of asexual parasites. Blood smears were examined by two readers and, in the case of discordant results, by a third reader. Discordant results were defined as a difference between the two readers in (a) *Plasmodium* species, (b) positive and negative, (c) with parasite >400/µL; if the higher count divided by the lower count was >2, (d) with parasite ≤400/µL; if the higher reading is >Log10 higher than the lower reading.

### Parasite genotyping

Molecular analysis was performed at the Molecular Biology laboratory of Centre Muraz of Bobo-Dioulasso, Burkina Faso. Dried blood spots from day 0 (pre-treatment) and from day of recurrent parasitaemia (post-treatment) during the 28 days follow-up were used for parasite genotyping. *Plasmodium falciparum* DNA was extracted from dried blood spots using QIamp DNA miniKit (Qiagen, Germany) following the manufacturer’s procedures and 80 µL of DNA template was obtained. DNA was either used immediately for a polymerase chain reaction (PCR) or stored at −20 °C for later analysis. A Nested PCR was used to analyse polymorphism in the merozoite surface protein *msp*-*1* and *msp*-*2* genes to distinguish between recrudescence and new infection [10].

### Sample size and data analysis

Sample size was calculated by assuming the efficacy of each study treatment to be at least 90 %. Under this assumption a sample of 155 patients per arm will be able to show at the 5 % significance level with 90 % power, that the difference in efficacy between treatments is not more than 10 %. Allowing for a loss to follow-up around 10 %, the final sample size per arm was 170 patients/year and then, 340 patients per arm for the 2 years. Data were double entered by two independent data clerks and a verification program was used to correct errors by referring to the original CRF. The primary treatment outcomes were adequate clinical and parasitological response (ACPR), early treatment failure (ETF), late clinical failure (LCF) and late parasitological failure (LPF). Total treatment failure (TTF) was determined as the sum of ETF, LCF and LPF [11]. Patients classified as treatment failures were given quinine (10 mg/kg orally three times a day for 7 days) and in case of severe malaria or danger signs parenteral quinine was administered. Treatment secondary outcome measures were the tolerability of the treatment that was assessed by the risk of occurrence of adverse events (AEs).

Statistical analysis was performed using STATA (IC), version 10.0 software. Differences between groups were assessed using the Chi square test for proportions and analysis of variances (ANOVA) for continuous normal distributed variables or the non-parametric Kruskal–Wallis to compare continuous not normally distributed variables. Kaplan–Meier product limit formula was used to estimate the risks of treatment failure and of new infections. A p-value of less than 0.05 was considered as statistically significant.

### Ethics

This was part of a larger study entitled ‘Pharmacovigilance for artemisinin-based combination treatments in Africa’ (ClinicalTrials.gov Identifier: NCT01232530). The study was conducted in accordance with the principle of good clinical practices (GCP). Before enrolment, each participant or parents/guardians signed informed consent form. In case of illiteracy, thumb-printed consent was obtained in the presence of unbiased witness. The study was reviewed and approved by Center Muraz Institutional Ethics Committee, Burkina Faso National Ethics Committee and the World Health Organization (WHO) Ethical Review Committee.

## Results

**Fig. 1:**
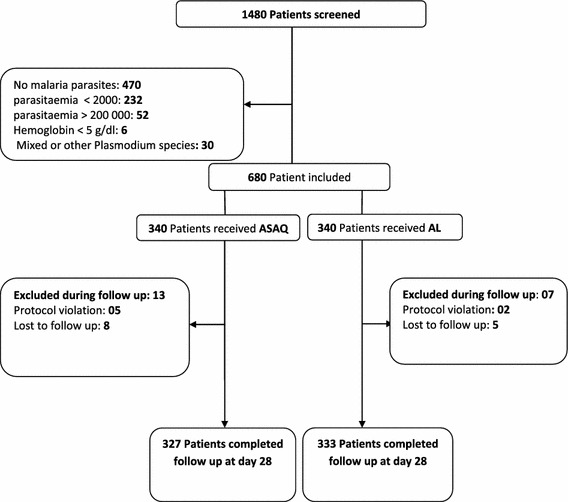
Trial profile. The figure shows the patients flow from the screening to the end of the follow-up time

A total of 1480 patients was screened to be included in the study. Among them, 1010 (68.2 %) patients were positive for malaria. Out of them, 680 patients were randomized to receive one of the two treatments. A total of 340 patients received ASAQ as treatment and 340 patients received AL. Twenty (20) patients (2.9 %) were excluded from the analysis and the reasons for exclusion and sampling plan are shown in trial profile (Fig. 1). A total of 496 (75.1 %) patients were children under 5 years old versus 164 (24.8 %) over 5 years of age. The baseline characteristics of patients included in the study are shown in Table 1. At recruitment, the two treatment groups were comparable in all characteristics except for the haemoglobin level, which was higher in AL group than in ASAQ group (p = 0.019).

**Table 1:** Baseline characteristics of included patients

Characteristics	AL, n = 340 (%)	ASAQ, n = 340 (%)	P-value
Site			
Nanoro, n (%)	176 (51.76)	176 (51.76)	–
Nazoanga, n (%)	164 (48.24)	164 (48.24)	
Sex			
Male, n (%)	183 (53.82)	182 (53.53)	0.939
Female, n (%)	157 (46.18)	158 (46.47)	
Age in years, median (p25–p75)	3.26 (1.70–4.98)	3.14 (1.61–5.05)	0.387
Weight in kg, median (p25–p75)	11 (9–14)	10 (9–14)	0.157
Mean temperature (SD)	38.41 (0.91)	38.47 (0.91)	0.381
Hemoglobin in g/dl, median (p25–p75)	9 (8–11)	9 (8–10)	0.019
GMPD (95 % CI)	30,529 (26,809–34,766)	30,763 (27,180–34,818)	0.933

By day 28 the ACPR was significantly higher in ASAQ group than in AL group before and after adjustment by PCR (Table 2). Before PCR adjustment ACPR in AL group was 47.7 versus 67.0 % in ASAQ group [risk difference = −0.19, 95 % CI (−26.62; −11.81), p < 0.00001]. The adjusted ACPR was 77.8 % in AL group versus 84.1 % in ASAQ group [risk difference = −6.32, 95 % CI (−12.29; −0.34), p = 0.0389]. Two ETF were found in AL group versus three in ASAQ treated patients.

**Table 2:** Primary treatment outcomes

Treatment outcome	AL, n = 333 (%)	ASAQ, n = 327 (%)	Difference (95 % CI)	P-value
PCR unadjusted				
ACPR	159 (47.8)	219 (67.0)	−0.19 (−26.62; −11.81)	<0.00001
TTF	174 (52.2)	108 (43.0)		
ETF	2 (0.6)	3 (0.9)		
LCF	47 (14.1)	18 (5.5)		
LPF	125 (37.5)	87 (26.6)		
PCR adjusted				
ACPR	259 (77.8)	275 (84.1)	−6.32 (−12.29; −0.34)	0.0389
TTF	74 (22.2)	52 (15.9)		
ETF	2 (0.6)	3 (0.9)		
LCF	22 (6.6)	11 (3.4)		
LPF	50 (15.0)	38 (11.6)		

In both AL and ASAQ groups, treatment outcomes were better in patients over 5 years old than in children under five before adjustment by PCR. Before adjustment, ACPR in AL group was 43.3 and 61.7 % in children under 5 years and patients over 5 years old respectively (p = 0.004). In ASAQ group ACPR before adjustment was 63.1 % in children under 5 years old versus 78.3 % in patients over 5 years of age (p = 0.013). After PCR adjustment, ACPR was higher in older patients than in children less than 5 years old in both treatment groups but the difference was not statistically significant (Table 3).

**Table 3:** Treatment outcomes by age group

Treatment	Treatment outcome	≤5 years, n = 496 (%)	>5 years, n = 164 (%)	P-value
AL	*PCR unadjusted*			
	ACPR	109 (43.3)	50 (61.7)	0.004
	TTF	143 (56.7)	31 (38.3)	
	*PCR adjusted*			
	ACPR	192 (76.2)	67 (82.7)	0.276
	TTF	60 (23.8)	14 (17.3)	
ASAQ	*PCR unadjusted*			
	ACPR	154 (63.1)	65 (78.3)	0.013
	TTF	90 (36.9)	18 (21.7)	
	*PCR adjusted*			
	ACPR	199 (81.6)	76 (91.6)	0.053
	TTF	45 (18.4)	7 (8.4)	

**Fig. 2:**
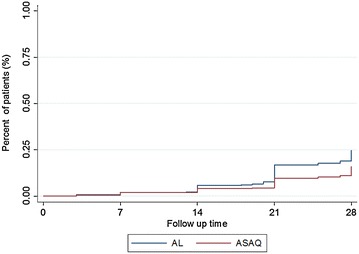
Kaplan–Meier survival curve of risk of recrudescence. The figure shows the percentage of patients with recrudescent infection during the 28 days follow-up. The *blue line* indicates patients treated with artemether–lumefantrine (AL), the *red line* indicates patients treated with artesunate–amodiaquine (ASAQ)

The risk of recrudescence in both AL and ASAQ group is represented in Fig. 2. The risk of recrudescence was higher in AL treated patients than in those receiving ASAQ (p = 0.0059). In both groups the risk of recrudescence became more observable after day 14.

**Fig. 3:**
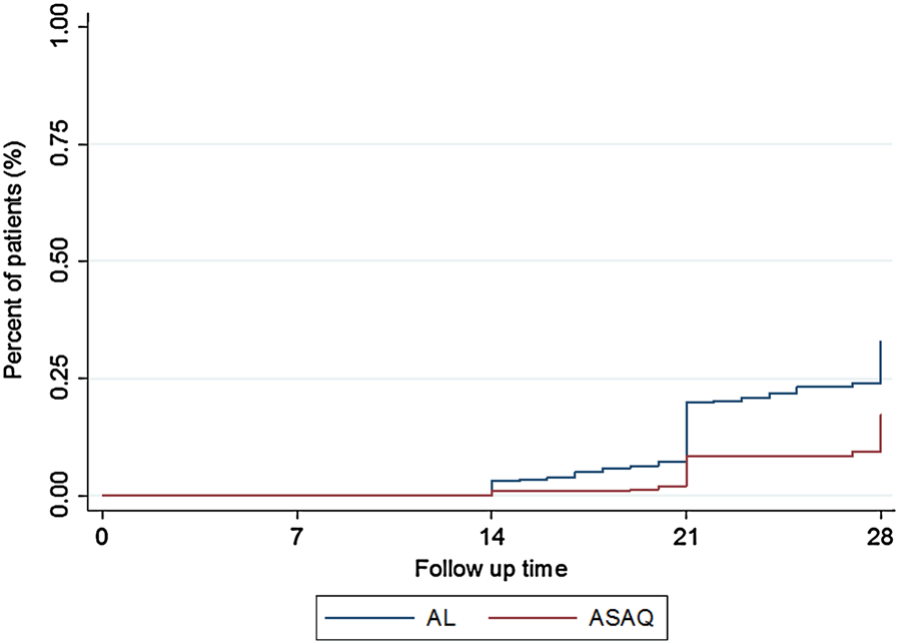
Kaplan–Meier survival curve of total risk of new infection. The figure shows the percentage of patients with new infection during the 28-day follow-up. The *blue line* indicates patients treated with artemether–lumefantrine (AL), the *red line* indicates patients treated with artesunate–amodiaquine (ASAQ)

Figure 3 represents the risk of new infection in each of the two treatment arms. The new infections start after day 14 and it was significantly higher in AL group than in ASAQ group.

Both AL and ASAQ treatment were well tolerated by the patients. An average of two AEs per patient was recorded in both AL and ASAQ treated patients. The most commonly observed AEs reported for each AL/ASAQ treatment were fever (n = 117/83) cough (n = 75/74), diarrhoea (n = 61/65), rhinitis (n = 19/20), anaemia (n = 17/17),vomiting (n = 11/20), abdominal pain (n = 15/11), anorexia (n = 9/12), conjunctivitis (n = 6/10), otitis (n = 9/5), headache (n = 7/7), and pruritus (n = 5/3). Six serious adverse events (SAE) were found in AL group versus four in ASAQ group and most of them were not linked to the treatment. The majority of AE was mild and was unlikely or definitely unrelated to the administrated treatment (Table 4).

**Table 4:** Safety information by treatment arm

Treatment	AL, n = 193	ASAQ, n = 188	P-value
Total number of adverse events (AE)	386	346	–
Mean AEs per patient	2	1.8	0.20
Severity of adverse events (%)			
Mild	83.4	84.7	0.642
Moderate	13.7	13.9	0.956
Serious	2.9	1.4	0.382
Relationship of events with the treatment (%)			
Definitely unrelated	29.5	30.3	0.810
Unlikely	54.2	55.2	0.774
Possible	12.7	10.7	0.401
Probable	3.6	3.8	0.926
Definitely related	0	0	–

## Discussion

Although numerous studies carried out in malaria endemic countries had shown good efficacy and safety of ACT for the treatment of uncomplicated malaria, the conditions of clinical trials do not fully reflect real field situation. Moreover, the assessment of the efficacy of the unsupervised therapy is of greatest interest in a context of home management of malaria by community medicines distributors. This study shows that the unsupervised cure rate after adjustment by day 28 is 77.8 and 84.1 %, respectively for AL and ASAQ, indicating a lowering of the cure rate when drug intake is not directly supervised. Similar findings have been observed in a study carried out on Gabonese children [12]. A recent study conducted in the same area has also shown a cure rate of 89.7 % for AL and 89.8 % for ASAQ after 42 days follow-up for the unsupervised malaria treatment [6]. However when drug administration is directly supervised a cure rate of about 90 % has always been observed [13, 14]. But as in this study drug intakes were not supervised and the assessment adherence was based on patient self-reporting on day 3, this lowering of the cure rate should be taken with caution. This constituted one of the limitations of our study because some studies have shown a difference in adherence rate obtained by the use of different methods and the assessment based on patients self-reporting seems not to be the most appropriate [15, 16]. Therefore, compliance to the treatment and above all, the preferable administration of AL with fatty food that could not be respected in real field conditions could explain the lowering of the cure rate. Thus the unsupervised therapy with AL results in lower plasma levels of lumefantrine with an increased risk for early infection [17]. Furthermore, vomiting was one of the commonest AEs observed in both AL and ASAQ treated patients. In the unsupervised condition it was difficult to obtain reliable information about time elapsed between drug intake and vomiting and this could affect the efficacy of the two drugs when it occurred a while after drugs intake. Moreover a recent ex vivo study conducted at the same area has shown a decrease in sensitivity of lumefantrine in the country and this could further explain this lowering of the cure rate [18]. In addition findings in a study carried out in Kenya indicated a decline in sensitivity of *P. falciparum* to ACT, in particular AL and dihydroartemisinin–piperaquine [19]. It is also known that some cases of artemisinin-resistance have been recorded in Asia, and a particular care for the wide spread use at community level should be taken for the preservation of the activity of these drugs [20]. Of particular interest in this study was the involvement of patients of all age groups and this shows that the parasitological cure rate in patients over 5 years is higher than for children under 5 years of age. This is not surprising and could be explained by the additive effect of the partially acquired immunity in aged patients living in endemic area due to frequent expositions to mosquito bites [21].

This study also shows that by day 28, over 50 % of treated patients had recurrent parasitaemia, most of them were re-infected with new *P. falciparum* strains. The risk of being re-infected is higher in AL treated patients than those receiving ASAQ and this starts after day 14 in both two study arms. The phenomenon of recurrent parasitaemia would increase the cost of malaria treatment and control and hence the use of a long half-life medicine would be necessary in such context.

Both AL and ASAQ were well-tolerated, similar to many other studies with AEs mostly mild and not linked to the administrated treatment [5, 22]. A limitation to this study was the challenge of getting reliable safety recall information from young children who constituted an important part of the study population.

## Conclusion

In a context where home management of malaria has been adopted as one of the main strategies for malaria control, the assessment of the effectiveness of unsupervised therapy is of fundamental importance. This study shows a lowering of the efficacy when drug intake is not directly supervised. The results reported here is worrying as both rates are lower than the critical threshold of 90 % required by the WHO to recommend the use of an anti-malarial drug in a treatment policy [23, 24]. Nevertheless, there is a need to conduct similar studies coupled with a collection of data on study participants’ adherence to treatment and diet habit to confirm these findings.

## Abbreviations

ACT: artemisinin combination therapy
AL: artemether–lumefantrine
ASAQ: artesunate–amodiaquine
*msp*-*1*: merozoite surface protein-1 gene
*msp*-*2*: merozoite surface protein-2 gene
PCR: polymerase chain reaction
ACPR: adequate clinical and parasitological response
ETF: early treatment failure
LCF: late clinical failure
LPF: late parasitological failure
TTF: total treatment failure
SAE: serious adverse event
DNA: de-oxy-ribonucleic-acid
GMPD: geometric mean parasite density
AE: adverse event
CRF: case record form

## References

[1] WHO. Antimalarial drug combination therapy: report of a technical consultation. Geneva: World Health Organization; 2001.

[2] Ministry of Health of Burkina Faso/PNLP. Directives Nationales pour la prise en charge du paludisme au Burkina Faso; 2006.

[3] Kobbe R, Klein P, Adjei S, Amemasor S, Thompson WN, Heidemann H (2008). A randomized trial on effectiveness of artemether–lumefantrine versus artesunate plus amodiaquine for unsupervised treatment of uncomplicated *Plasmodium falciparum* malaria in Ghanaian children. Malar J.

[4] Bassat Q, Mulenga M, Tinto H, Piola P, Borrmann S, Menéndez C (2009). Dihydroartemisinin–piperaquine and artemether–lumefantrine for treating uncomplicated malaria in African children: a randomised, non-inferiority trial. PLoS One.

[5] Meremikwu M, Alaribe A, Ejemot R, Oyo-Ita A, Ekenjoku J, Nwachukwu C (2006). Artemether–lumefantrine versus artesunate plus amodiaquine for treating uncomplicated childhood malaria in Nigeria: randomized controlled trial. Malar J.

[6] Tinto H, Diallo S, Zongo I, Guiraud I, Valea I, Kazienga A (2014). Effectiveness of artesunate–amodiaquine vs. artemether–lumefantrine for the treatment of uncomplicated falciparum malaria in Nanoro, Burkina Faso: a noninferiority randomised trial. Trop Med Int Health.

[7] Ministry of Health of Burkina Faso/DSN. Acting plan 2014 of the Nanoro Sanitary District; 2013.

[8] Derra K, Rouamba E, Kazienga A, Ouedraogo S, Tahita MC, Sorgho H (2012). Profile: Nanoro health and demographic surveillance system. Int J Epidemiol.

[9] WHO. Toxicity grading scale for determining the severity of adverse events. World Health Organization; 2003.

[10] Ranford-Cartwright L, Taylor J, Umasunthar T, Taylor L, Babiker H, Lell B (1997). Molecular analysis of recrudescent parasites in a *Plasmodium falciparum* drug efficacy trial in Gabon. Trans R Soc Trop Med Hyg.

[11] WHO. Assessment of therapeutic efficacy of antimalarial drugs for uncomplicated falciparum malaria. Geneva: World Health Organization; 2003.

[12] Oyakhirome S, Pötschke M, Schwarz NG, Dörnemann J, Laengin M, Salazar CO (2007). Artesunate–amodiaquine combination therapy for falciparum malaria in young Gabonese children. Malar J.

[13] Zongo I, Dorsey G, Rouamba N, Dokomajilar C, Séré Y, Rosenthal PJ (2007). Randomized comparison of amodiaquine plus sulfadoxine–pyrimethamine, artemether–lumefantrine, and dihydroartemisinin–piperaquine for the treatment of uncomplicated *Plasmodium falciparum* malaria in Burkina Faso. Clin Infect Dis.

[14] Ndiaye JL, Randrianarivelojosia M, Sagara I, Brasseur P, Ndiaye I, Faye B (2009). Randomized, multicentre assessment of the efficacy and safety of ASAQ—a fixed-dose artesunate–amodiaquine combination therapy in the treatment of uncomplicated *Plasmodium falciparum* malaria. Malar J.

[15] Adams A, Soumerai SB, Lomas J, Ross-Degnan D (1999). Evidence of self-report bias in assessing adherence to guidelines. Int J Qual Health Care.

[16] Bell DJ, Wootton D, Mukaka M, Montgomery J, Kayange N, Chimpeni P (2009). Measurement of adherence, drug concentrations and the effectiveness of artemether–lumefantrine, chlorproguanil–dapsone or sulphadoxine–pyrimethamine in the treatment of uncomplicated malaria in Malawi. Malar J.

[17] White NJ, van Vugt M, Ezzet FD (1999). Clinical pharmacokinetics and pharmacodynamics of artemether–lumefantrine. Clin Pharmacokinet.

[18] Tinto H, Bonkian LN, Nana LA, Yerbanga I, Lingani M, Kazienga A (2014). Ex vivo anti-malarial drugs sensitivity profile of *Plasmodium falciparum* field isolates from Burkina Faso five years after the national policy change. Malar J.

[19] Borrmann S, Sasi P, Mwai L, Bashraheil M, Abdallah A, Muriithi S (2011). Declining responsiveness of *Plasmodium falciparum* infections to artemisinin-based combination treatments on the Kenyan coast. PLoS One.

[20] Phyo AP, Nkhoma S, Stepniewska K, Ashley EA, Nair S, McGready R (2012). Emergence of artemisinin-resistant malaria on the western border of Thailand: a longitudinal study. Lancet.

[21] Ladeia-Andrade S, Ferreira MU, de Carvalho ME, Curado I, Coura JR (2009). Age-dependent acquisition of protective immunity to malaria in riverine populations of the Amazon Basin of Brazil. Am J Trop Med Hyg.

[22] Brasseur P, Vaillant MT, Olliaro PL (2012). Anti-malarial drug safety information obtained through routine monitoring in a rural district of South-Western Senegal. Malar J.

[23] WHO. Guidelines for the treatment of malaria. Geneva: World Health Organization; 2010. 25473692

[24] WHO. Global report on antimalaria drug efficacy and drug resistance: 2000–2010. Geneva: World Health Organization; 2000.

